# Supplementary material for: Effective Unidirectional Wetting of Liquids on Multi-Gradient, Bio-Inspired Surfaces Fabricated by 3D Printing and Surface Modification
Source: Polymers (Basel). 2024 Jun 30;16(13):1874. doi: 10.3390/polym16131874 (PMC11244379; doi:10.3390/polym16131874)
Supplement: Supplementary file 1 [file polymers-16-01874-s001.zip › polymers-3031907-supplementary.pdf]

Supplementary materials

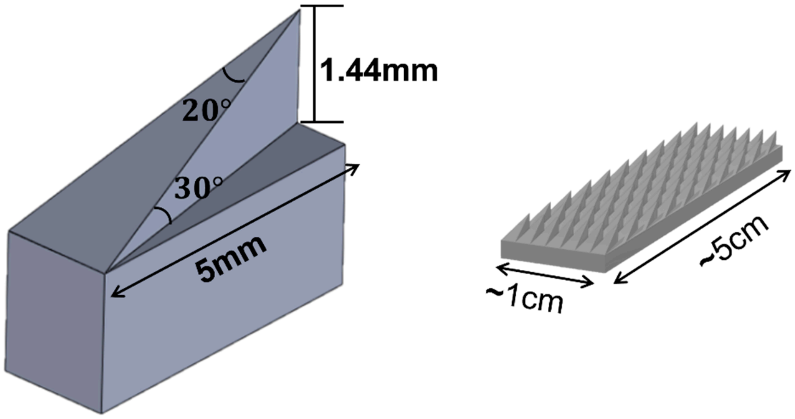

Figure S1. The design parameters of tetrahedron pattern.

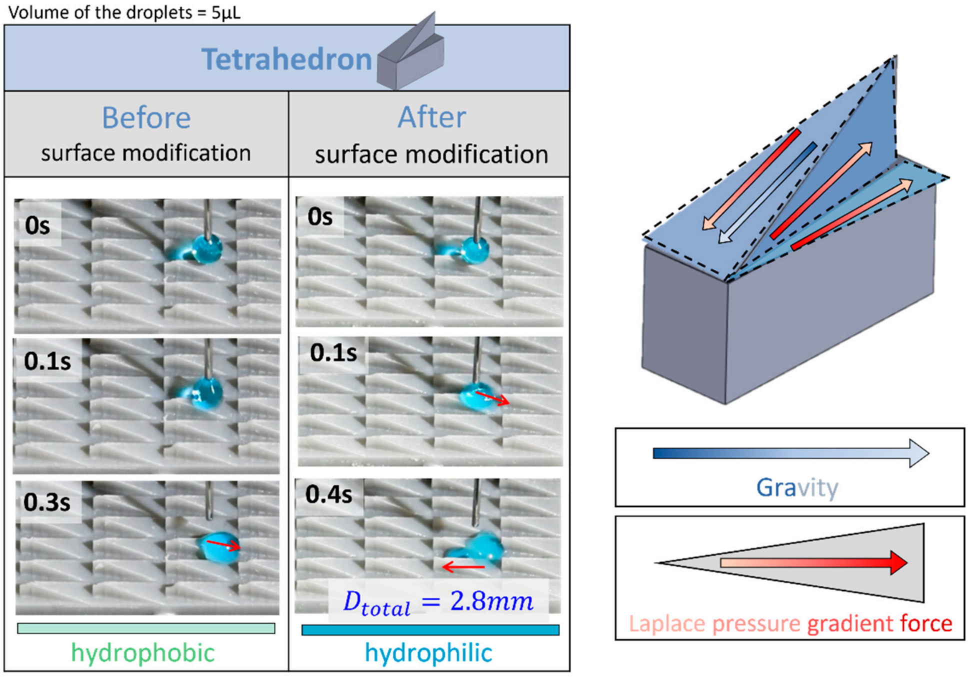

Figure S2. The dynamic wetting behavior of a water droplet on 3D printed ABS-like samples with (a) untreated; (b) modified tetrahedron patterns. The lighter blue indicates the surface is hydrophobic (CA~100°). The darker blue indicates the surface is hydrophilic (CA~10°).

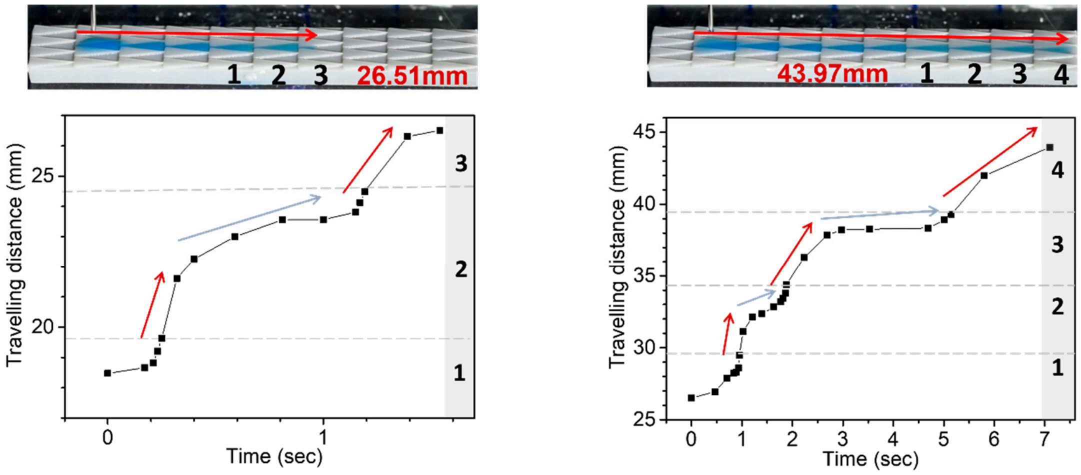

(a) (b)

Figure S3. (a) Plot of the traveling distance versus time on the multi-gradient surface. The numbers 1-3 labeled are associated with each triangular prism position. (b) Plot of the traveling distance versus time on the multi-gradient surface. The numbers 1-4 labeled are associated with each triangular prism groove

(a) (b)

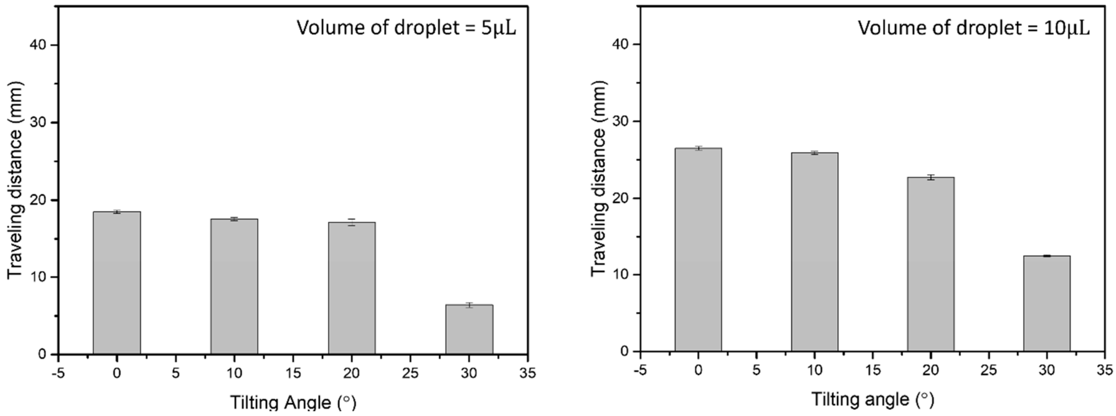

(c)

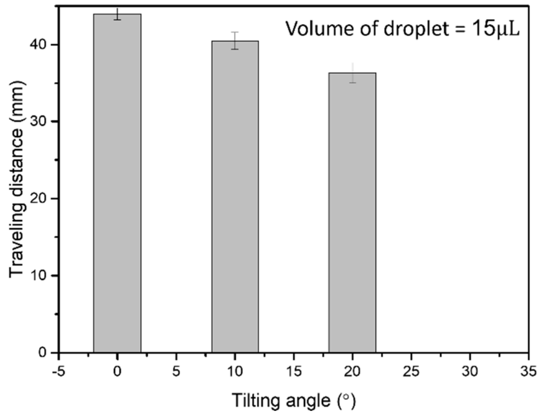

Figure S4. Plot of the traveling distance versus the tilt angle. The volume of the water droplet is (a) 5μl; (b) 10μl; and (c) 15μl.
